# Muscle synergies in Parkinson’s disease before and after the deep brain stimulation of the bilateral subthalamic nucleus

**DOI:** 10.1038/s41598-023-34151-6

**Published:** 2023-04-28

**Authors:** Marco Ghislieri, Michele Lanotte, Marco Knaflitz, Laura Rizzi, Valentina Agostini

**Affiliations:** 1 Department of Electronics and Telecommunications, Politecnico di Torino, 10129 Turin, Italy; 2 PolitoBIOMed Lab, Politecnico di Torino, 10129 Turin, Italy; 3 Department of Neuroscience “Rita Levi Montalcini”, University of Turin, 10126 Turin, Italy; 4 AOU Città della Salute e della Scienza di Torino, 10126 Turin, Italy

**Keywords:** Parkinson's disease, Motor control, Biomedical engineering, Parkinson's disease

## Abstract

The aim of this study is to quantitatively assess motor control changes in Parkinson’s disease (PD) patients after bilateral deep brain stimulation of the subthalamic nucleus (STN-DBS), based on a novel muscle synergy evaluation approach. A group of 20 PD patients evaluated at baseline (before surgery, T_0_), at 3 months (T_1_), and at 12 months (T_2_) after STN-DBS surgery, as well as a group of 20 age-matched healthy control subjects, underwent an instrumented gait analysis, including surface electromyography recordings from 12 muscles. A smaller number of muscle synergies was found in PD patients (4 muscle synergies, at each time point) compared to control subjects (5 muscle synergies). The neuromuscular robustness of PD patients—that at T_0_ was smaller with respect to controls (PD T_0_: 69.3 ± 2.2% vs. Controls: 77.6 ± 1.8%, *p* = 0.004)—increased at T_1_ (75.8 ± 1.8%), becoming not different from that of controls at T_2_ (77.5 ± 1.9%). The muscle synergies analysis may offer clinicians new knowledge on the neuromuscular structure underlying PD motor types of behavior and how they can improve after electroceutical STN-DBS therapy.

## Introduction

Parkinson’s disease (PD) is one of the most common neurodegenerative disorders and it is mainly characterized by a degeneration of the pars compacta of the substantia nigra and of the striatum pathway. As a result, basal ganglia nuclei increase their inhibitory projections to cortical areas and to the brainstem, leading to abnormal activation of different subcortical dopaminergic and non-dopaminergic structures (i.e. cerebellum, subthalamic nucleus, …) that are involved in movements. Motor symptoms such as bradykinesia, rigidity, tremor, and postural balance disorder, as well as non-motor symptoms such as cognitive decline and depression, represent the principal clinical manifestations of PD^1^. Gait disorders play an outstanding role in PD, becoming one of the major sources of disability with the progression of the disease. Locomotion is typically slow and shuffling, stride length is shortened, velocity is reduced despite a normal cadence, and fall risk is higher compared to age-matched healthy subjects^2^.

High-frequency bilateral deep brain stimulation (DBS) of the subthalamic nucleus (STN) is a well-established surgical therapy for patients affected by advanced PD and motor complications that cannot be adequately managed through medications^3^. STN-DBS has been demonstrated as an effective treatment for advanced PD patients, leading to a good control of all the PD cardinal symptoms (i.e. rigidity, bradykinesia, and tremor) and the drug-induced motor complications (i.e. motor fluctuations and dyskinesias)^4–6^. Pre-surgery Unified Parkinson’s disease rating scale (UPDRS) motor scores may often be improved by two-thirds in response to bilateral STN-DBS. Unfortunately, the ability to detect gait changes through the UPDRS gait item is limited. On the other hand, studies performed with instrumented movement analysis highlighted significant improvements after bilateral STN-DBS neurosurgery on anticipatory postural adjustments before gait initiation, kinematics and kinetics of lower limb joints during steady-state walking, and upper limb locomotor synergies, which are commonly affected in the advanced stage of PD both off and on anti-Parkinsonian medications. From this perspective, STN-DBS appears to provide good relief for key motor symptoms of advanced PD^7^.

While instrumented movement analysis in general (i.e. through tools such as the instrumented Timed-Up-and-Go test (iTUG)^8^), and in particular gait analysis^9^ already widely proved its validity in providing an objective assessment of the global PD functional performance, there is a lack of studies specifically addressing patients’ motor coordination during locomotion. Although neuronal dysfunction is at the origin of PD motor deficits, neural activity during locomotion is not directly measurable. As a consequence, the large majority of clinical tests focus on assessing motor outputs either at the behavioral level or providing kinetic, kinematic, and spatio-temporal gait parameters. However, the alteration of these parameters could be the result of multiple distinct neural abnormalities with very different muscle coordination patterns^10^.

In this perspective, new approaches are needed to quantitatively and non-invasively assess motor control strategies during gait in patients affected by advanced PD before and after STN-DBS, to enhance the knowledge of the effects of high-frequency stimulation on the locomotor control system. Since both basal ganglia and cerebellum play a key role in the supraspinal modulation of the temporal profile of muscle activations^11^, muscle synergy analysis may represent a powerful tool to quantitatively and non-invasively investigate the pathophysiological mechanism underlying PD motor symptoms and assess the effects of pharmacological and non-pharmacological (i.e. electroceutical) therapies.

A better understanding of the functional deficits in the impaired PD nervous system may be obtained by recording and analyzing the activity of the main muscles involved in locomotion, through surface electromyography (sEMG). Unfortunately, sEMG datasets usually present high intra- and inter-subject variability and are often difficult to interpret^12,13^, frequently limiting their clinical usefulness^14^. However, specific computational analyses, based on the muscle synergy theory, can be applied to sEMG datasets to reveal underlying motor control patterns and strategies that are thought to reflect neural function^15,16^. According to the muscle synergy theory, the central nervous system (CNS) may control the activation levels and the synchrony of all the muscles involved in a specific motor task through a small number of low-dimensional elements (called *muscle synergies*), rather than independently controlling every single muscle. For example, several previously published studies demonstrated that human locomotion can be modeled, both in healthy and pathological conditions, by a small set of muscle synergies characterized by specific and clear biomechanical functions^17–20^. Depending on individual characteristics, as well as on the type and number of muscles acquired and on the sEMG pre-processing techniques applied and the factorization algorithm implemented, the number of muscle synergies characterizing human locomotion commonly ranges between 4 and 5^17–22^.

Even if to date no consensus has been reached on the neural origin of the muscle synergies^16^, several studies have supported this theory both in healthy and pathological conditions (i.e. patients suffering from a wide variety of different neurological and orthopedic disorders altering their gait pattern), allowing for an objective and non-invasive evaluation of the motor control strategies adopted by the CNS during different motor tasks. From this perspective, the muscle synergy theory is a valuable candidate to assess changes in motor control strategies in response to high-frequency STN-DBS in advanced PD patients. To date, only a few studies investigated muscle synergies in patients with advanced PD during locomotion to assess pathophysiological mechanisms underlying motor symptoms and the effect of dopaminergic therapy^23–25^. In the study by Rodriguez et al*.*^23^, a fewer number of muscle synergies was found in PD patients than in healthy controls, revealing a reduced neuromuscular complexity during gait compared to the age-matched healthy subjects. Roemmich et al.^24^ demonstrated that CNS modular organization (assessed through the number, the composition, and the timing of muscle synergies) is not responsive to dopaminergic medications in patients with PD during both postural and locomotion tasks. Post-rehabilitative improvements in muscle synergy consistency, generalizability, and distinctness were reported by Allen et al*.*^25^, while no changes in the number of muscle synergies were detected after dance-based rehabilitation. However, due to different experimental approaches used to extract muscle synergies (i.e. number of muscles acquired, sEMG preprocessing techniques, number of gait cycles considered, factorization algorithms, …), small sample size (10 PD patients on average), and heterogeneity (i.e. poor clinical and behavioral characterization) of the patient cohorts, many inconsistencies and a lack in the comparability of the muscle synergy results can be noticed among those studies^23,24,26–30^.

To date, no studies have been published investigating the short- and long-term effects of high-frequency STN stimulation on the modular organization of the CNS during walking. Only one study by Falaki et al*.*^31^ reported that STN-DBS does not influence muscle synergies during postural tasks. Hence, there is a lack of studies focusing on muscle synergies analysis of PD patients during locomotion, and on how PD patients’ motor coordination can be possibly improved after STN-DBS. This study aims at documenting the motor control alterations of PD patients, and their possible improvements following the bilateral neurostimulation therapy, by evaluating the patients’ locomotor performance before the STN-DBS surgery and after it (3 and 12 months after DBS), through instrumented gait and muscle synergy analyses. Primary outcomes will be the number of muscle synergies expressed during locomotion, their composition, and their robustness.

## Results

The demographic and anthropometric characteristics of the PD (before DBS and 12 months after DBS) and healthy populations enrolled in the study are detailed in Table 1.

**Table 1:** Demographic and anthropometric characteristics of PD and healthy populations.

		Sex	Age (years)	Weight (kg)	Height (m)	UPDRS-III (Best-on condition)	H&R (Best-on condition)	Disease duration (years)
PDs	Before DBS	7 F, 13 M	58.1 ± 2.0	75.5 ± 2.9	1.73 ± 0.02	18.0 ± 1.6***	I–II	11.7 ± 0.8
	12-mo after DBS	N/A	N/A	N/A	N/A	10.5 ± 1.0***	I–III	12.9 ± 0.8
Controls		8 F, 12 M	57.3 ± 2.1	74.2 ± 4.6	1.70 ± 0.02	N/A	N/A	N/A

Parameters’ values are reported as mean ± standard error over the sample population.

*M* males, *F* females, *UPDRS*-*III* unified Parkinson’s disease rating motor subscale, *H&Y* Hoehn and Yahr scale, *N/A* not applicable or not assessed.

Statistically significant differences are represented through asterisks (***p < 0.001).

No statistically significant differences were detected between the PD (at baseline) and control groups for age, weight, and height. On average, PD patients clinically improved their motor performance after DBS surgery: the UPDRS-III motor scale showed a baseline value of 18.0 ± 1.6 points (mean value ± standard error) as measured before DBS, that significantly decreased to 10.5 ± 1.0 points at 12 months after DBS (*p* = 0.0001, Hedges’ $g$ statistic: $g$ = 1.22).

The motor performance of subjects was quantitatively evaluated during locomotion through instrumented gait analysis. PD patients were evaluated at three different time points: before DBS (baseline T_0_), 3 months after DBS (T_1_), and 12 months after DBS (T_2_).

Gait parameters and the main muscle synergy outcome measures of PD patients (at T_0_, T_1_, and T_2_) and controls are reported in Table 2, with the assumption of homogeneity of variances checked through the Levene’s $F$ test ($\alpha$ = 0.05) and the statistically significant differences among groups tested through the repeated measures one-way ANOVA ($\alpha$ = 0.05).

**Table 2:** Gait and muscle synergy analysis parameters for PD patients and controls during locomotion.

	PD patients			Control subjects	Levene’s $F$ test	1-way ANOVA ($p$-value)
	Before DBS (T_0_)	3-mo after DBS (T_1_)	12-mo after DBS (T_2_)			
Gait parameters						
Walking speed (m/s)	0.96 ± 0.04	0.95 ± 0.03	0.89 ± 0.04**	1.05 ± 0.04**	0.09	**0.04**
Cadence (cycles/min)	55.5 ± 1.3	56.5 ± 0.9	55.7 ± 1.2	54.0 ± 0.8	0.09	0.42
Muscle synergy outcome measures						
Number of muscle synergies	4.3 ± 0.1**	4.3 ± 0.1^ϮϮ^	4.2 ± 0.1^‡‡^	4.8 ± 0.1**^,ϮϮ,‡‡^	0.56	**0.006**
Neuromuscular robustness (%)	69.3 ± 2.2*^,ϮϮ,‡‡^	75.8 ± 1.8*	77.5 ± 1.9^ϮϮ^	77.6 ± 1.8^‡‡^	0.75	**0.013**

Parameters’ values are reported as mean ± standard error over the sample population.

Groups: PD patients before DBS (T_0_), PD patients at 3 months after DBS (T_1_), PD patients at 12 months after DBS (T_2_), and controls.

Statistically significant differences among groups are represented through asterisks (*p < 0.05; **p < 0.01), daggers (^Ϯ^*p* < 0.05; ^ϮϮ^*p* < 0.01), and double daggers (^‡^*p* < 0.05; ^‡‡^*p* < 0.01).

Significant values are in bold.

There was a slightly significant difference in walking speed among Groups (1-way ANOVA: $F$(3, 75) = 2.87, *p* = 0.04), while no differences were observed in cadence (1-way ANOVA: $F$(3, 76) = 0.94, *p* = 0.42). The Fisher’s least significant difference (LSD) test for multiple comparisons revealed that the walking speed was significantly lower in PD patients at 12 months after DBS (T_2_) compared to controls (*p* = 0.005, $g$ = 0.91).

There was a statistically significant difference in the number of muscle synergies among groups (1-way ANOVA: $F$(3, 72) = 4.55, *p* = 0.006). The number of muscle synergies was significantly smaller in PD patients (at each of the three time points considered) compared to controls. Indeed, Fisher’s LSD test for multiple comparisons revealed that the number of muscle synergies was diminished in PD patients at T_0_ (*p* = 0.005, $g$ = 0.90), at T_1_ (*p* = 0.005, $g$ = 0.90), and at T_2_ (*p* = 0.002, $g$ = 1.05) compared to control subjects.

There was a statistically significant difference in the neuromuscular robustness (1-way ANOVA: F(3, 68) = 3.89, *p* = 0.013) among Groups. The robustness of PDs at baseline (T_0_) was smaller than that of controls, but then it increased after DBS surgery, becoming not different from that of controls at 12 months after DBS surgery (T_2_). In particular, the Fisher’s LSD test for multiple comparisons revealed lower values of robustness in PD patients at T_0_ compared to T_1_ (*p* = 0.021, *g* = 0.75), at T_0_ compared to T_2_ (*p* = 0.005, *g* = 0.94), and at T_0_ compared to controls (*p* = 0.004, *g* = 0.97). No statistically significant difference was detected between PD patients at T_2_ compared to controls. A deeper analysis of this outcome measure (named “*neuromuscular robustness*” or simply “*robustness*” of motor control) will be provided after the description of the muscle synergy composition.

**Figure 1:**
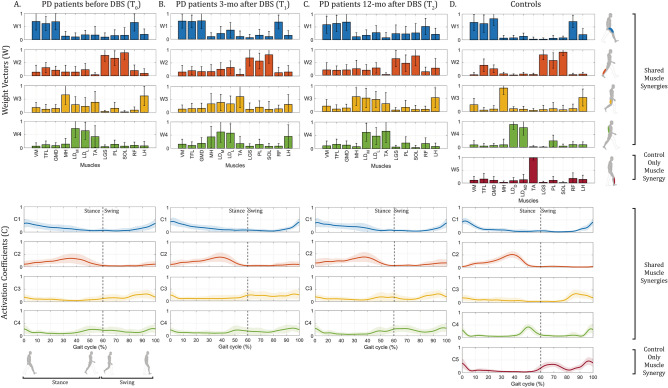
Comparison of muscle synergies extracted during walking from (**A**) PD patients before DBS (T_0_), (**B**) PD patients 3 months after DBS (T_1_), (**C**) PD patients 12 months after DBS (T_2_), and (**D**) controls. For each synergy *k*, the weight vector ${W}_{k}$ (bars showing the muscles’ contribution levels) and the corresponding activation coefficient ${C(t)}_{k}$ (time profile of the neural command) are displayed with the same color (average and standard deviation across the population are displayed). Vertical dotted lines represent the transition between the stance and the swing phase of the gait cycle. Muscle abbreviations: *VM*  vastus medialis, *TFL*  tensor fasciae latae, *GMD*  gluteus medius, *MH*  medial hamstring, *LD*  longissimus dorsii (for PD patients: *LD*_*M*_  ≡ *LD* of the more affected side, *LD*_*L*_ ≡ *LD* of the less affected side; for controls: *LD*_*D*_ ≡ *LD* of the dominant side, *LD*_*ND*_ ≡ *LD* of the non-dominant side), *TA*  tibialis anterior, *LGS*  lateral gastrocnemius, *PL*  peroneus longus, *SOL*  soleus, *RF*  rectus femoris, and *LH*  lateral hamstring.

Considering the muscle synergy composition, Fig. 1 represents the average weight vectors and activation coefficients of the muscle synergies expressed during the walking task of PD patients before DBS (baseline T_0_), 3 months after DBS (T_1_), 12 months after DBS (T_2_), and of control subjects. From a biomechanical point of view, a specific motor function can be associated with each muscle synergy by observing the prevailing muscle contribution levels ($W$) and the time profile of the activation coefficients ($C$). Considering control subjects (Fig. 1D), five different biomechanical functions can be identified: three functions are mainly related to the generation of the cyclic pattern of gait, while the remaining two functions are related to body stabilization and dynamic postural control. More specifically, the motor functions that were identified are the following:

1. First synergy (in blue) is mainly defined by the activation of the *VM*, *TFL*, *GMD*, and *RF* muscles at the beginning and at the end of the gait cycle. This synergy is used to stabilize the hip joint and keep the knee partially extended during the heel strike and the load acceptance phase;
2. Second synergy (in orange) is mainly defined by the activation of the *LGS*, *PL*, and *SOL* muscles during mid and terminal stance phases to generate propulsion;
3. Third synergy (in yellow) is mainly defined by the activation of the *MH* and *LH* muscles during the late swing phase to decelerate the leg and the foot;
4. Fourth synergy (in green) is mainly defined by the activation of the *LD*_*D*_ and *LD*_*ND*_ muscles to control the trunk position in the frontal plane at the heel strike of the homo- and contra-lateral foot;
5. Fifth synergy (in red) is mainly defined by the activation of the *TA* muscle during the swing phase to decelerate the foot during the first rocker and to control forefoot clearance from the floor.

PD patients, at all time points (T_0_, T_1_ and T_2_), expressed four muscle synergies during locomotion, while controls were better represented by five muscle synergies. Fewer synergies were needed to account for muscle activation patterns in PD patients before DBS (Fig. 1A), 3 months (Fig. 1B), and 12 months after DBS (Fig. 1C) compared to controls.

**Figure 2:**
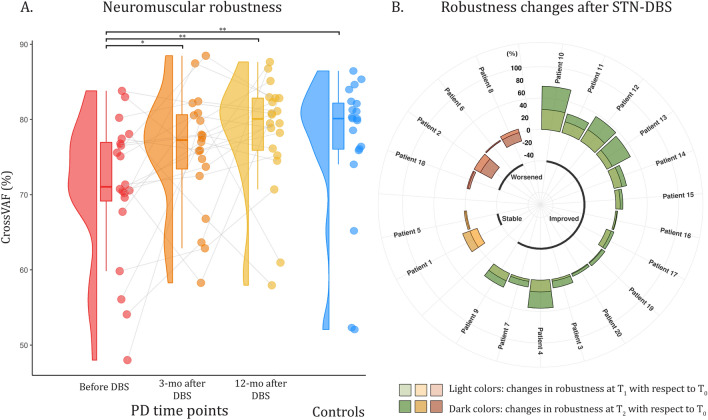
Panel (**A**) Raincloud plots of the neuromuscular robustness computed by means of the cross variance accounted For (${Cross}_{VAF}$) from PD patients before DBS (T_0_, in red), 3 months after DBS (T_1_, in orange), 12 months after DBS (T_2_, in yellow), and controls (in blue). For each group, the raincloud plot shows, sequentially, the data distribution (split-half violin plot), a standard visualization of central tendency with a boxplot (representing minimum, 25th percentile, median, 75th percentile, and maximum), and raw jittered data points of each specific individual (scatter plot). Panel (**B**) Circular bar diagram of the relative changes in muscle synergy robustness at 3 months after DBS with respect to baseline (T_1_–T_0_), and at 12 months after DBS with respect to baseline (T_2_–T_0_), for each PD patient. Green bars represent PD patients that improved the robustness of their motor control after DBS neurosurgery, yellow bars PD patients that did not change their robustness, and red bars PD patients that worsened their robustness after DBS neurosurgery. Single (*) and double (**) asterisks represent statistically significant differences with *p*-values lower than 0.05 and 0.01, respectively.

Concerning neuromuscular robustness, Fig. 2A represents the raincloud plots of the robustness computed through the ${Cross}_{VAF}$ from each of the tested groups. At baseline (T_0_), the robustness of PD patients is reduced with respect to that of controls, but then PD robustness increases after DBS, already at T_1_ (with respect to T_0_) and even more evidently at T_2_ (with respect to T_0_), becoming not different from that of controls at T_2_. The longitudinal assessment of each PD patient can be appreciated from the trend lines connecting the colored circular dots (each dot represents a single patient at a specific time point). Longitudinal modifications in neuromuscular robustness are further analyzed for each specific PD patient. In Fig. 2B, changes in robustness at 3 months after DBS compared to baseline (T_1_–T_0_), and at 12 months after DBS with respect to baseline (T_2_–T_0_) are represented for each PD patient. Positive values of robustness changes (T_1_–T_0_ > 0 or T_2_–T_0_ > 0) are representative of an improvement of the motor control robustness after DBS neurosurgery, while negative values describe a worsening in motor control robustness. Depending on the robustness changes, PD patients were clustered into three different groups: (a) patients who overall revealed positive robustness changes after STN stimulation were clustered in the “Improved” group, (b) patients who overall revealed no robustness changes were clustered in the “Stable” group, and (c) patients who overall revealed negative robustness changes were clustered in the “Worsened” group. Fourteen PD patients out of 20 (Patient 3, 4, 7, 9, 10, 11, 12, 13, 14, 15, 16, 17, 19, and 20) were clustered in the “Improved” group. Overall, their robustness increased 3 months after DBS neurosurgery, and further increased 12 months after DBS, revealing an incremental improvement in robustness. Two patients (Patient 1 and 5) were clustered in the “Stable” group. Overall, no changes in neuromuscular robustness were detected 12 months after DBS compared to the baseline condition. The remaining 4 PD patients (Patient 2, 6, 8, and 18) were clustered in the “Worsened” group. Overall, they worsened their motor control robustness 3 months after DBS and further worsened 12 months after DBS.

**Figure 3:**
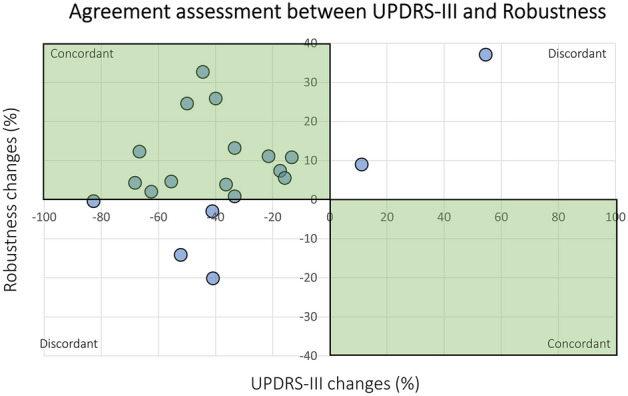
Agreement between UPDRS-III changes (*x*-axis) and neuromuscular robustness changes (*y*-axis). The relative changes at 12 months after DBS with respect to baseline are represented (T_2_–T_0_). The quadrants showing concordance are highlighted in green.

The agreement assessment between the UPDRS-III and the neuromuscular robustness is shown in Fig. 3, considering the relative changes at 12 months after DBS with respect to baseline (T_2_–T_0_). It can be observed that 14 out of 20 patients improved their performance both in terms of the motor clinical score (as expressed by UPDRS-III) and the newly introduced biomarker neuromuscular robustness (top-left quadrant). Moreover, there are no patients that worsened both UPDRS-III and neuromuscular robustness (bottom-right quadrant). On the other hand, there are 4 patients that improved their UPDRS-III, but not their robustness (bottom-left quadrant), and 2 patients that worsened their UPDRS-III, but improved their robustness (top-right quadrant). Kendall rank correlation coefficient (Kendall’s τ coefficient) is equal to 0.24, revealing a moderate agreement between the clinical score and the robustness parameter.

## Discussion

To the best of the authors’ knowledge, this is the first study that non-invasively and quantitatively evaluates changes in locomotor strategies after high-frequency STN-DBS neurosurgery in advanced PD patients through the analysis of the muscle synergies. Moreover, it is the first study to show that neuromuscular robustness may act as a meaningful and predictive biomarker of improved motor performance in individuals with PD after STN-DBS. From a neurophysiological point of view, this motor biomarker can be related to the variability (low robustness) or the stability (high robustness) of the motor control along the walking task.

To increase the statistical power of the study, a sample population of 20 PD patients was assessed at three different time points and compared against an age- and gender-matched sample population of 20 healthy volunteers. PD patients were enrolled in the study following strict inclusion criteria based on standardized clinical scales (such as the UPDRS-III and H&R scale for the clinical assessment and the MMSE and FAB for the neuropsychological and behavioral assessment) to accurately select a homogenous cohort. The same neuropsychological and behavioral assessment was performed also on the control subjects to exclude subjects affected by cognitive and/or psychiatric diseases.

In this study, PD patients were tested always during their optimal pharmacological condition (T_0_: ON medication, T_1_: ON medication + DBS ON, T_2_: ON medication + DBS ON). This was chosen to compare the patients’ best-ON condition at the 3 different time points. This was also justified by the fact that for many of our patients it would have been very difficult, or even impossible, to autonomously perform a 5-min walking task in their OFF medication state. In addition, we did not expect to find differences between ON and OFF medication states from the point of view of muscle synergies, from what is already known from the literature^24,26,27,32^. In the perspective of testing PD patients during their best-ON condition, the stimulation parameters (other than the stimulation frequency) were tailored to the specific needs of each patient for obtaining their “best” condition.

In accordance with previously published studies, a reduction in the number of muscle synergies in individuals with PD compared to age-matched healthy subjects was demonstrated in the present study, suggesting an overall reduction in the complexity of the motor control system often associated with poorer motor performance. Indeed, in motor control systems characterized by a lower number of muscle synergies, the ability of the CNS to independently control muscle synergies deputed to different biomechanical functions is often strongly reduced, revealing a lack of modular independence and therefore a reduced motor control complexity. In PD patients, the reduced number of muscle synergies observed suggests that some of the biomechanical functions identified in the controls are merged into a single function. This is related to an increased muscle coactivation (appreciable from Weight Vectors) and a reduced independence of neural control signals (appreciable from Activation Coefficients). More specifically, muscle synergies related to body stabilization and dynamic postural control (i.e. the fourth and the fifth muscle synergies described for controls) appear to be the most affected by this muscle synergy merging observed in PD patients, suggesting a reduced ability to independently control muscle synergies deputed to those biomechanical functions. A reduced muscle synergy number, and activation coefficients less independent from each other (i.e. a reduced sparseness of synergy timing profiles), compared to age-matched healthy controls were demonstrated also in other studies involving stroke survivors^33^.

The analysis of the muscle synergy number in PD patients at different time points revealed that the number of muscle synergies necessary to properly describe the walking task is not sufficient to fully assess motor performance improvements after STN-DBS neurosurgery in individuals affected by PD. To further support this finding, a previous study by Roemmich et al*.*^24^ demonstrated that the muscle synergy number is not affected by dopaminergic medications commonly used to improve motor performance, suggesting that some aspects of the neuromuscular control may not be covered by the number of muscle synergies. Consistently, our results demonstrated that there was no increase in the average number of muscle synergies expressed by the enrolled PD individuals after STN-DBS (PD before DBS: 4.3 ± 0.1 synergies, PD at 3 months after DBS: 4.3 ± 0.1 synergies, PD at 12 months after DBS: 4.2 ± 0.1 synergies) despite clinical improvements in disease symptoms (i.e. an average reduction in UPDRS-III motor score at 12 months after DBS neurosurgery). Furthermore, four PD individuals surprisingly decreased their muscle synergy number 12 months after neurosurgery.

The muscle synergy extraction process used in this study allows for assessing the robustness of the modular organization of the CNS over the whole duration of the 5-min walking task by computing muscle synergies on different 10-gait-cycle time-epochs for each participant. Neuromuscular robustness was used to assess within-subject variability (also known as intra-subject variability) in locomotor strategies. In particular, an increased robustness is representative of a diminished intra-subject variability of the motor control. The results of this study revealed a significant increase in the neuromuscular robustness in the locomotion of PD patients, already at 3 months and even more clearly at 12 months after DBS, compared to PD patients before DBS. In other words, an improved robustness might be interpreted as a more repeatable motor control along the 5-min walk, provided by smoother and less variable neural commands during the task execution. From the clinical point of view, an enhanced robustness, that can be read as a higher intra-subject locomotion regularity, can be hypothesized to have an impact in decreasing the fall risk of PD patients. Unfortunately, at the moment, we do not have data to support this hypothesis. Future studies might investigate this aspect.

The finding of an increased neuromuscular robustness is in line with previous studies that demonstrated that trial-by-trial variability of sEMG burst patterns observed during reaching tasks in PD patients can be highly improved through the stimulation of the subthalamic nucleus^34^.

In this study, we provide evidence that neuromuscular robustness is a more sensitive outcome measure than the muscle synergy number in highlighting short- and long-term motor performance improvements after high-frequency STN-DBS in PD patients. Indeed, an increase in the neuromuscular robustness was assessed, on average, 3 months and 12 months after STN-DBS, revealing an effect of the high-frequency stimulation on the variability of the neural control during walking. Moreover, PD patients 12 months after neurosurgery achieved a neuromuscular robustness statistically similar to the one observed in control subjects.

A limitation of this study is the lack of a longitudinal evaluation of PD patients without STN-DBS to be used as a reference population. A second limitation is the fact that no clinical scores (UPDRS-III and H&Y) were collected from PD patients at 3 months after DBS surgery. According to the experimental protocol approved by the local ethical committee, the clinical scores were assessed only at baseline (T_0_) and 1 year after neurosurgery (T_2_). Another limitation is the lack of an analysis of the biomechanical changes and gait spatio-temporal parameters associated with the reduced number of synergies and lower robustness in PD patients. Future studies might specifically investigate these aspects. Furthermore, incorporating a wider set of parameters extracted from the weight vectors and the activation coefficients will also be necessary to determine whether there are other parameters capable of covering additional aspects of the neuromuscular control of individuals affected by advanced PD.

## Online methods

### Sample population

A sample population composed by 20 patients affected by advanced PD (gender: 7 females and 13 males; age: 58.1 ± 2.0 years; weight: 75.5 ± 2.9 kg; height: 1.73 ± 0.02 m) and 20 age- and gender-matched healthy controls (gender: 8 females and 12 males; age: 57.3 ± 2.1 years; body mass: 74.2 ± 4.6 kg; height: 1.70 ± 0.02 m) was enrolled in the present study. PD volunteers were enrolled at the Stereotactic and Functional Neurosurgery Unit of the University of Turin (Turin, Italy) among those patients eligible for high-frequency (130 Hz) bilateral STN-DBS neurosurgery. More specifically, the inclusion criteria for the PD patients were the following:

- Diagnosis of Parkinson’s disease according to the UK Brain Bank principles;
- Good levodopa response;
- Treatment-resistant motor fluctuation and dyskinesia;
- Age at DBS surgery under 70 years;
- Absence of dementia or severe cognitive impairment, psychiatric or behavioral disturbances;
- Absence of freezing of gait and postural instability unresponsive to pharmacological treatment;
- Absence of abnormalities at cerebral MRI or relevant condition that may increase surgical risk;
- Ability to independently walk for a few minutes without using walking aids or external support during the best-ON time window.

PD patients characterized by the presence of co-morbidities potentially affecting gait performance (i.e. knee or hip prostheses) were excluded from the present study. Age- and gender-matched patients’ caregivers were enrolled as healthy controls, excluding those reporting a history of neurological or musculoskeletal disorders affecting gait.

Neuropsychological and behavioral assessments were performed on both pathological and healthy volunteers through the mini-mental state examination (MMSE)^35^, a widely used screening measure of global cognitive functioning, and the Frontal assessment battery (FAB)^36^. In this study, only PD patients and healthy controls achieving an MMSE score equal to or higher than 25 (i.e. normal cognitive function) and a FAB score equal to or higher than 15.3 were included.

This study was reviewed and approved by the Ethics Committee of A.O.U. Città della Salute e della Scienza di Torino – A.O. Ordine Mauriziano – A.S.L. “Città di Torino” (No. 0092029 approved on September 11, 2018). Following the Declaration of Helsinki, all participants gave written informed consent for the experimental procedure.

### Experimental design

**Figure 4:**
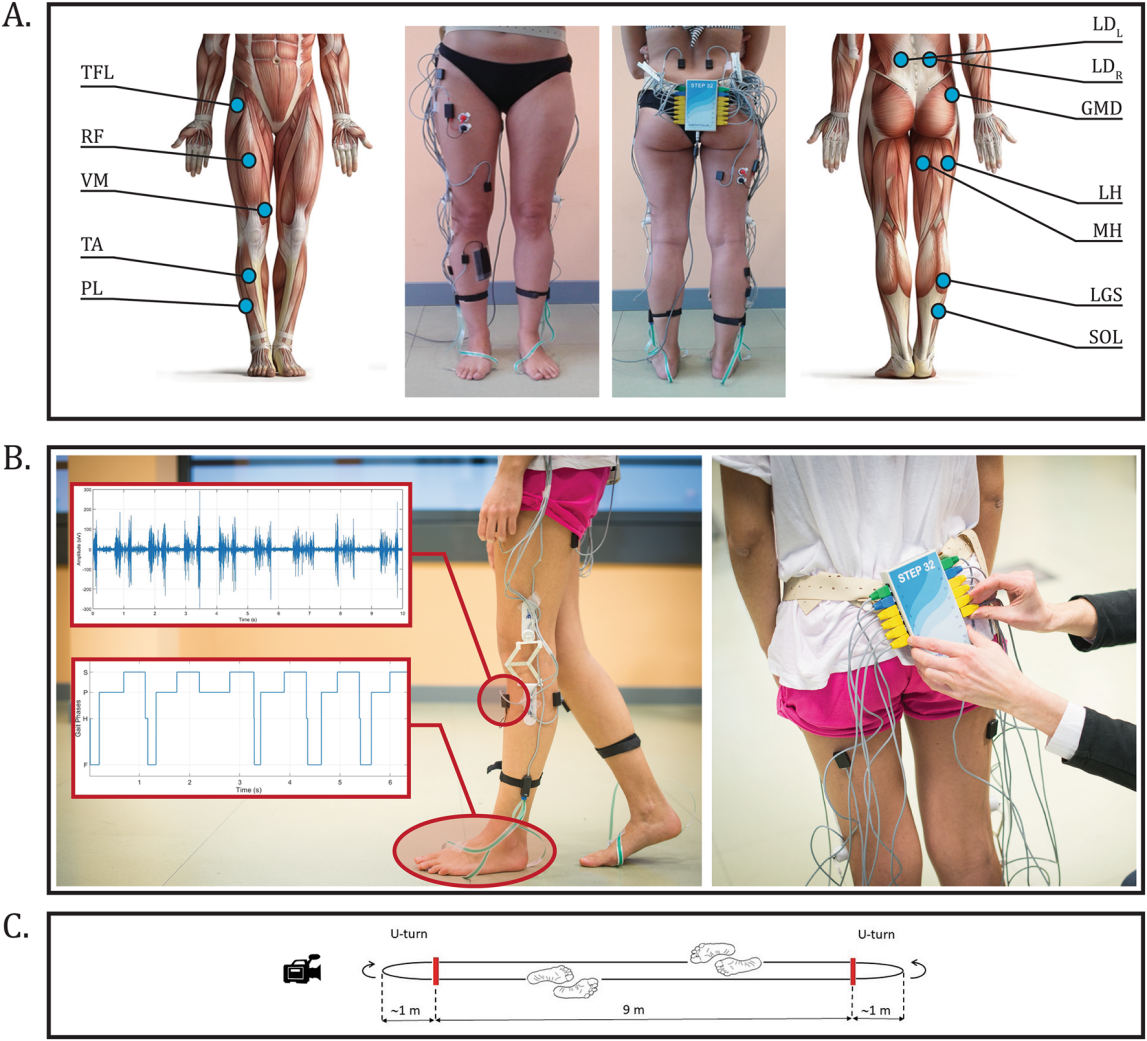
Experimental design and data recordings. (**A**) Sensor placement for a healthy representative subject of the sample population. SEMG active probes are placed over twelve muscles of the dominant (or more affected) lower limb and the trunk (bilaterally). Foot-switches are placed beneath the heel, the first, and the fifth metatarsal heads to detect gait phases. Electro-goniometers are placed on the lateral aspect of the knee joint, bilaterally. (**B**) Examples of sEMG (upper panel) and foot-floor contact (lower panel) signals acquired through the system STEP32 (Medical Technology, Turin, Italy). (**C**) Schematic representation of the walking path. Subject walked barefoot back and forth along a 9-m straight path, for approximately 5 min at a self-selected pace.

Volunteers walked barefoot, at self-selected speed, for approximately 5 min back and forth over a 9-m straight walkway. PD patients were tested during their best-ON pharmacological condition and performed the overground walking task at three different time points: before the STN-DBS neurosurgery (before DBS, baseline T_0_), 3 months after the STN-DBS neurosurgery (3-mo after DBS, T_1_), and 12 months after the STN-DBS neurosurgery (12-mo after DBS, T_2_) to assess both short- and long-term changes in motor control strategies due to high-frequency stimulation. Healthy controls, instead, performed the overground walking task only once. For both pathological and healthy volunteers, recordings began after a preliminary habituation phase. All the experimental sessions were performed at the Motion Analysis Laboratory of the Polito^BIO^Med Lab (Politecnico di Torino, Turin, Italy). Figure 4C schematically represents the walking path.

### Data recordings

Surface electromyographic (sEMG) signals were recorded from twelve trunk and lower limb muscles through the STEP32 system (Medical Technology, Turin, Italy) for clinical gait analysis (weight: 0.3 kg, size: 80 mm × 30 mm × 150 mm)^12,37–39^. The most affected side of PD patients (9 left and 11 right lower limbs) and the dominant side of the healthy participants (2 left and 18 right lower limbs) were considered. SEMG signals were recorded through surface active probes^40,41^ (configuration: single differential; Ag-disks diameter: 4 mm; interelectrode distance: 12 mm; size: 19 mm × 17 mm × 7 mm) at a sampling frequency of 2000 Hz, converted by a 12-bit analog-to-digital converter, and sent to a PC for real-time representation. The recorded trunk and lower-limb muscles included: left and right Longissimus Dorsii (*LD*), Tensor Fasciae Latae (*TFL*), Gluteus Medius (*GMD*), Rectus Femoris (*RF*), Lateral and Medial Hamstrings (*LH* and *MH*, respectively), Vastus Medialis (*VM*), Lateral Gastrocnemius (*LGS*), Peroneus Longus (*PL*), Soleus (*SOL*), and Tibialis Anterior (*TA*)^26,42–44^.

In addition, gait phases were simultaneously recorded through three foot-switches (size: 10 mm × 10 mm × 0.5 mm; activation force: 3 N) placed bilaterally under the barefoot sole in correspondence of the heel, the first, and the fifth metatarsal heads. Foot-switches were used to detect foot-floor contact events and segment the gait cycles as described by Agostini et al*.*^45^. Electro-goniometers were also positioned on the lateral aspect of the knee joint, bilaterally. Figure 4A and B show an example of sEMG probes and foot-switches placement for a representative healthy participant and an example of signals acquired during walking, respectively.

All of the acquired data were processed offline on MATLAB® release 2021b (MathWorks Inc., Natick, USA) through custom routines.

### Muscle synergy extraction

#### SEMG pre-processing

Foot-switch signals were used to automatically time-segment sEMG signals into gait cycles based on the foot-floor contact sequences^45^. Only gait cycles belonging to the straight path were considered, discarding those corresponding to curved trajectories and direction changes at the beginning and at the end of the walkway (including also deceleration before and acceleration after a direction change)^44^.

First, sEMG signals were bandpass filtered between 10 and 500 Hz through a 2nd zero-lag IIR Butterworth filter. Then, sEMG signals were high-pass filtered (8th order zero-lag IIR Butterworth digital filter with a cut-off frequency of 35 Hz), rectified, and low-pass filtered (4th order zero-lag IIR Butterworth digital filter with a cut-off frequency of 12 Hz) according to Ghislieri et al*.*^42,46^. Each sEMG envelope was first amplitude-normalized to the global maximum of each acquired muscle for each participant^41^ and then time-normalized to 1000 samples per gait cycle^33^. Each gait cycle was divided into 600 samples for the stance phase and 400 samples for the swing phase to avoid biases due to the absolute durations of the gait phases and to help assessing the temporal contribution of the different muscle synergies to each gait cycle sub-phase. The normalized sEMG envelopes were then grouped into time epochs of 10 concatenated gait cycles (i.e. gait cycles from 1 to 10 belong to time-epoch 1, gait cycles from 11 to 20 belong to time-epoch 2, …), allowing for muscle synergy assessment over the whole duration of the task^22,42^.

#### Muscle synergy model and factorization algorithm

In this study, the modular organization of the CNS was modeled through the “*time-invariant*” muscle synergy model^47^. According to this model, the pre-processed sEMG signals can be described as the linear combination of two low-dimensional components: *(i)* the time-independent muscle synergy weights ($W$) and *(ii)* the time-dependent activation coefficients ($C$) as detailed in Eq. (1):

1 $$M\left(t\right)={\sum }_{k=1}^{N}{C(t)}_{k} \cdot {W}_{k}+e,$$

where $M\left(t\right)$ is the matrix containing the pre-processed sEMG signals acquired from the observed muscles at a specific time point $t$, ${W}_{k}$ is a time-independent vector of non-negative weights representing the spatial information of the motor control $k$, ${C(t)}_{k}$ is a time-dependent set of non-negative muscle activation coefficients representing the temporal information of the motor control $k$ at a specific time point $t$, $N$ is the number of muscle synergies necessary to reconstruct the original data, and $e$ is the model reconstruction error.

The Non-Negative Matrix Factorization (NNMF) algorithm^48^ was used to extract a set of $N$ time-invariant muscle synergies which minimize the reconstruction error from each 10-gait-cycle time-epoch. This algorithm uses a multiplicative update rule for the optimization of the non-negative weight vectors and activation coefficients as described by Lee et al.^49^. The MATLAB® function *“nnmf”* was used to factorize the pre-processed sEMG data, setting the number of replicates equal to 15, the maximum number of iterations equal to 1000, and the function and search tolerance equal to 1e^-6^ according to Ghislieri et al*.*^42,46^. To improve the performance of the NNMF algorithm in reconstructing the original sEMG signals, a sparseness constraint was imposed in the first initialization of the muscle synergy weights^50^, while a standard random initialization (values randomly selected from a uniform distribution in the range [0, 1]) was performed for the activation coefficients.

Different NNMF solutions were tested by running several times the algorithm on the same sEMG data changing the muscle synergy number ($N$) between 1 and 12 (i.e. the total number of muscles acquired). For each number of muscle synergies, the ${R}^{2}$ similarity between the original and the reconstructed sEMG data was computed to assess the quality of the reconstruction. Consequently, the number of muscle synergies required to properly reconstruct the original sEMG signals was defined as the number of synergies at which the ${R}^{2}$ vs. number of synergies curve achieves the highest curvature (called “elbow” criterion). More specifically, the curvature was calculated for every three consecutive points of the ${R}^{2}$ vs. number of synergies curve (i.e. first considering points 1, 2, and 3, then points 2, 3, and 4, …)^51^. The “elbow” criterion was implemented to avoid setting arbitrary cut-off thresholds on the model reconstruction quality (such as 90% of the fraction of data variation accounted for by the muscle synergy model)^51^.

Before muscle synergy analysis, muscle synergies were sorted in the same order for each 10-gait-cycle time-epoch and each volunteer by applying the $k$-means clustering algorithm to the muscle synergy weight vectors^20,42^. The MATLAB® function *“kmeans”* was used to sort muscle synergies setting the following input parameters: $N$ as number of $k$-means clusters, 1000 as maximum number of iterations, 15 as number of replicates, squared cosine as distance metric, and $k$-means^++^ as algorithm to choose the $k$ initial cluster centers^42^. The non-negative activation coefficients ($C$) were then sorted consequently.

### Muscle synergy analysis

Muscle synergies extracted from PD patients (before DBS, 3 months after DBS, and 12 months after DBS) and healthy participants were quantitatively compared in terms of *(i)* muscle synergy number (${N}_{syn}$) and *(ii)* muscle synergy robustness (${Cross}_{VAF}$). Muscle synergy compositions (i.e. weight vectors and activation coefficients), instead, were qualitatively compared.

#### Number of muscle synergies (${\mathrm{N}}_{\mathrm{syn}}$)

The number of muscle synergies needed to properly reconstruct the original sEMG signals belonging to each 10-gait-cycle time-epoch (${N}_{subgroup}$) was selected by choosing the number of synergies at which the ${R}^{2}$ vs. number of synergies curve achieves the highest curvature (“elbow” criterion)^51^. Then, for each subject, the final number of synergies (${N}_{syn}$) was computed as the mode of the numbers of muscle synergies extracted from each 10-gait-cycle time-epoch to proceed with the statistical analysis^42^. ${N}_{syn}$ can assume values between 1 and 12 (i.e. the total number of muscles recorded). This parameter has been recently proposed in the literature to assess the motor control complexity and its reduction in cases of pathological conditions^23,33,52,53^. Higher values of muscle synergy number have been demonstrated to model more complex motor control strategies, while a reduction in motor control complexity (and hence in the muscle synergy number) has been assessed in several pathological conditions^23,33,52–54^.

#### Neuromuscular robustness (${\mathrm{Cross}}_{\mathrm{VAF}}$)

To assess the neuromuscular robustness over time (along the walking task), we introduce a parameter describing how well a time epoch containing 10 gait cycles can be reconstructed by a muscle synergy model computed from a different 10-gait-cycle time-epoch.

Neuromuscular robustness among different 10-gait-cycle epochs (*i* and *j*) from the same walking task, was assessed through the cross-variance accounted for (${{\varvec{C}}{\varvec{r}}{\varvec{o}}{\varvec{s}}{\varvec{s}}}_{{\varvec{V}}{\varvec{A}}{\varvec{F}}}$)^42,55^ defined as in Eq. (2):

2 $${Cross}_{VAF}^{i,j}=\left(1-\frac{{\sum }_{k=1}^{m}{({M}_{k}^{i}-{M}_{k}^{j,R})}^{2}}{{\sum }_{k=1}^{m}{({M}_{k}^{i})}^{2}}\right)\cdot 100,$$

where ${M}_{k}^{i}$ describe the original, and ${M}_{k}^{j,R}$ the reconstructed sEMG envelopes of the $k$-muscle for the $i$*-* and $j$*-th* 10-gait-cycle time-epochs, respectively. This parameter assesses how well the muscle synergy model extracted from $i$*-th* time-epoch reconstructs the sEMG signals that belong to the $j$*-th* time-epoch as described by Ghislieri et al*.*^42,43^. It can assume values between 0 and 100%, corresponding to a low and high correlation between the original and reconstructed sEMG signals belonging to different time-epochs, respectively. Increased robustness of muscle synergies across different 10-gait-cycle time-epochs has been demonstrated to act as a meaningful marker of improved motor performance and neural control^25^.

### Statistical analysis

Before performing ANOVA and *post-hoc* analyses, Levene’s $F$ test was used to test the homogeneity of variances of all analyzed parameter distributions^56^. One-way analysis of variance (ANOVA) for repeated measures followed by *post-hoc* analysis with Fisher’s Least Significant Difference (LSD) adjustment for multiple comparisons was conducted for each extracted parameter to compare PD patients’ gait data and muscle synergy parameters before and after STN-DBS surgical therapy (3 and 12 months after DBS) with those of healthy participants.

All the analyses were performed with the significance level ($\alpha$) set equal to 0.05. All analyzed parameters were expressed as estimated mean values and standard errors. The effect size of the statistically significant differences was calculated through the Hedges’ $g$ statistic, including correction for small sample sizes (sample size ≤ 20)^57^. According to the study by Hedges et al*.*^57^, a $g$ (absolute) value of approximately 0.2, 0.5, and 0.8 are considered as small, medium, and large effect size, respectively. The agreement between neuromuscular robustness and the clinical UPDRS-III score was assessed through the Kendall rank correlation coefficient (τ) considering the relative changes at 12 months after DBS with respect to baseline (τ < 0.2: weak agreement; 0.2 < τ < 0.3: moderate agreement; τ > 0.3: strong agreement).

The statistical analysis was carried out using SPSS Statistical Software, version 28.0 (SPSS Inc., Chicago, IL).

### Institutional review board statement

The study was conducted according to the guidelines of the Declaration of Helsinki and approved by the Ethics Committee of A.O.U. Città della Salute e della Scienza di Torino – A.O. Ordine Mauriziano – A.S.L. “Città di Torino” (No. 0092029 approved on September 11, 2018).

### Informed consent

Informed consent was obtained from all subjects involved in the study before data acquisition.

## Data Availability

Data and materials presented in this study are available from the corresponding author upon reasonable request.

## Abbreviations

ANOVA: Analysis of variance
CNS: Central nervous system
DBS: Deep brain stimulation
GMD: Gluteus medius
H&Y: Hoehn and Yahr scale
LD_D_: Dominant side longissimus dorsii
LD_L_: Less affected side longissimus dorsii
LD_M_: More affected side longissimus dorsii
LD_ND_: Non-dominant side longissimus dorsii
LGS: Lateral gastrocnemius
LH: Lateral hamstrings
LSD: Fisher’s least significant difference
MH: Medial hamstrings
NNMF: Non-negative matrix factorization
PD: Parkinson’s disease
PL: Peroneus longus
RF: Rectus femoris
SOL: Soleus
STN: Sub-thalamic nucleus
TA: Tibialis anterior
TFL: Tensor fasciae latae
UPDRS: Unified Parkinson’s disease rating scale
VAF: Variance accounted for
VM: Vastus medialis
